# Role of agile leadership in managing inter-role conflicts for a satisfying job and life during COVID-19 in a VUCA world

**DOI:** 10.3389/fpsyg.2022.979792

**Published:** 2022-10-18

**Authors:** Saima Aftab, Komal Khalid, Ajmal Waheed, Asma Aftab, Aisha Adnan

**Affiliations:** ^1^Department of Management Sciences, University of Wah, Wah Cantt, Pakistan; ^2^Foundation University, Islamabad, Pakistan; ^3^Department of Human Resource Management, Faculty of Economics and Administration, King Abdulaziz University, Jeddah, Saudi Arabia; ^4^Department of Business Administration, Foundation University, Islamabad, Pakistan; ^5^Department of Ophthalmology, Wah Medical College, Wah Cantt, Pakistan; ^6^Boots UK Limited, Nottingham, United Kingdom

**Keywords:** COVID-19, agile leadership, role stress, work–family conflicts, family–work conflicts, life satisfaction, job satisfaction

## Abstract

This study investigated how agile leadership played its role in managing inter-role conflicts during the chaotic period of the COVID-19 pandemic. The COVID-19 pandemic was much more than the survival of the fittest and coming out of it alive. Organizations were under immense pressure to resume their normal operations in not-so-normal situations. This period of turmoil and agony brought a broad array of inter-role conflicts, which posed challenges for leaders to manage them effectively. The satisfaction at job and the satisfaction in life were the two most important endeavors for the employees to fight. This study explores how leadership agility helped employees manage their work–family and family–work conflicts, consequently impacting life satisfaction and job satisfaction simultaneously. Moreover, role ambiguity, role conflict, and role overload are important intervening role stress factors that impact inter-role conflict management. So, role stress is a moderating factor in the direct relationship between agile leadership and inter-role conflict. This is a two-phased time lag study with a quantitative design for data collection. The first phase of data collection comprises of analyzing the impact of agile leadership on inter-role conflict management, keeping in view the intervening impact of role stress. The second data collection phase examines how inter-role conflicts impacted life satisfaction and job satisfaction during COVID-19. The data were collected from faculty working in higher education institutions in Pakistan, as the education industry was the second major sector that was affected because of COVID-19 after the health care industry. This research found that agile leadership plays a significant role in determining job satisfaction and life satisfaction. Agile leadership during the COVID-19 pandemic helped to manage work–family (AgileL -> WFC -> JS β = 0.1020, *p* = 0.0112 and AgileL -> WFC -> LS β = 0.1361, *p* = 0.0014) and family–work conflicts (AgileL -> FWC -> JS β = 0.1598, *p* = 0.0017 and AgileL -> FWC -> LS β = 0.1160, *p* = 0.0093) and reduce role stress. Future researchers might include marital satisfaction, as the inter-role conflicts highly impacted marital satisfaction and resultant imbalances among dual-earning couples. Comparative studies in this regard, explaining how dual-earning couples managed to sustain marital health and the role of leadership in developed and developing countries would be enlightening.

## Introduction

The corporate world is currently dealing with a very volatile, uncertain, complex, and ambiguous scenario, referred to as a volatile, uncertain, complex and ambiguous (VUCA) world (Setiawati, 2021). The COVID-19 pandemic has wreaked havoc on education systems worldwide, affecting approximately 1.6 billion students in over 200 nations. More than 94% of the world’s student population has been affected by the closure of schools, institutions, and other learning facilities (Zheng et al., 2022). The pandemic has affected the faculty in these educational institutes as much as it has affected students worldwide, resulting in significant changes in every part of personal and professional lives (Pokhrel and Chhetri, 2021; Zheng et al., 2022). As many “new normal” emerged (Zacher and Rudolph, 2021), a pandora box of new challenges for employees in terms of change adaption, job satisfaction, and a significant impact on employee well-being and happiness is unlocked (Nemteanu et al., 2021; Antino et al., 2022). The impacts of the COVID-19 pandemic, regarded as one of humanity’s worst calamities in recent history, were initially studied in terms of human health, but the epidemic also had sociocultural, economic, and psychological consequences (Zacher and Rudolph, 2021). The closure of institutes and dual role maintenance at home triggered work–family conflicts at the most, as escalated role expectations triggered stress (Hong et al., 2021; Antino et al., 2022) along with increasing familial or financial obligations (Bozkurt et al., 2020). The COVID-19 pandemic has increased the difficulties the workplace is already facing (Ratten, 2020), making the distinction between the home and the office hazier than ever before (Chang et al., 2010). So, work–family conflicts and family–work conflicts are the most common byproducts of COVID-19 pandemic work practices.

The pandemic-induced unfavorable scenarios had an important psychological outcome in the form of a reduction in overall life satisfaction (Karakose et al., 2021). Individuals’ preferred work–life is more than just a job; it is a phenomenon that incorporates their aims, aspirations, and talents, and it is a key aspect in assuring their happiness in life (Samanta et al., 2021). As millennials currently make up the majority of the workforce worldwide (Naderi and Van Steenburg, 2018) who are labeled as “Me, me, me generation” (Wood, 2019), give prime importance to the ideology of personal well-being and work–life balance (Roestenburg, 2020). Individuals’ life satisfaction and job satisfaction are affected by direct or indirect stress sources from the teaching profession (Çelik and Üstüner, 2018). Research suggests that an individual’s work–life balance, work, and family conflicts are associated with job satisfaction, life satisfaction, and family satisfaction (Allen et al., 2010). The unprecedented demands from education sector employees to facilitate the smooth conduct of educational operations prompted a rapid increase in work burdens, pushing employees to work beyond office hours with resource constraints resulting in conflicts and ambiguities regarding the roles performed. Role stress results from a mismatch between an individual’s understanding of a given role and their current role while executing the assigned tasks (Pereira et al., 2022). According to boundary theory presented by Ashforth et al. (2000), when people think there is role overload in a specific domain, they would reallocate their resources among other roles to complete the interdomain transition resulting in role conflicts (Mellner et al., 2021). So, the role of leaders in chaos plays a vital role in managing employee job satisfaction and life satisfaction for which maintaining work and family conflicts should be the prime focus of today’s pandemic-induced HR strategies within organizations.

The COVID-19 pandemic drove the globe to respond in novel and unorthodox ways (Nissim and Simon, 2020). Although the pandemic has brought many misfortunes, agile leadership has emerged as a new norm. Agile leadership has become an essential organizational component in times of acute crisis (Al Fannah et al., 2020). It was the need of the time to reach a quick solution, one that relied on flexibility and agility in unexpected circumstances. It was especially important to retain and continue organizational activities and to manage the transition from a professional educational environment to a system of distant study, teaching, practice, and administration (Nissim and Simon, 2020). So, agile leadership practices aiding flexibility, adoption, innovation, and responsiveness helped organizations steer through these traumatic times of pandemic, especially in the education sector where an emergency was declared in the name of “emergency remote education (ERE)” as an obligation rather than an option (Bozkurt et al., 2020; Eichenauer et al., 2022) and the governments and educational institutes were struggling to ensure quality education without much disruption. Keeping in view the severity of the issue, no such study exists in the extant literature which examined the role of agile leadership and its subjective behavioral outcomes regarding inter-role conflict management effecting job and life satisfaction at the same time, during the times of pandemic.

**FIGURE 1:**
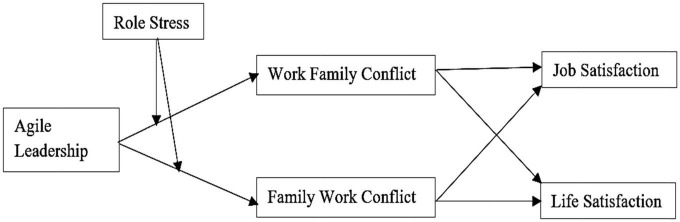
Proposed conceptual model.

So, this study is inspired by the situation of Pakistani higher education institutes, which collectively struggled very hard to fulfill their responsibilities on two critical fronts, i.e., toward their students as well as toward their employees. This study focuses on studying the role of agile leadership in managing the conflicts and behaviors of education sector employees during the COVID-19 pandemic. So, it contributes theoretically to the extant literature by examining how agile leadership practices in the education sector can help enhance job satisfaction and life satisfaction of teaching faculty by mitigating the spillover effects of work–family conflicts and family–work conflicts during the COVID-19 pandemic, keeping in view the intervening character of role stress which triggers conflicts either way. Figure 1 below presents the proposed model of the study, explaining the conceptual relationships among the study variables.

## Literature review

This study is based on the Work–Home Resources (W-HR) model presented by Ten Brummelhuis and Bakker (2012). The theory postulates that contextual work resources enhance family outcomes through increases in other resource categories, such as capital and close-to-home personal resources. Resources augment personal resources at work and home. These individual assets can be used to enrich outcomes at home and work. Work–home enrichment can be thought of as the process through which personal resources are developed using contextual resources from both the home and work. After that, performance in the other domain is made easier by the personal resources developed in each domain. A good mood and improved self-esteem, for instance, may result from the spouse’s emotional support (a contextual resource). A strong and resilient work attitude or even better job performance may result from using such personal resources at work. The contextual resource support (leadership) from organizational culture and context may help enhance the resources and provide meaning to work and life.

This study is supported by W-HR resources model by explaining that agile leadership is an organizational contextual resource supportive element that can enable the personal resource replenishment (time, energy, and moods) resulting in lesser family and work conflicting outcomes. Resultantly, lesser the work and family conflicts higher will be the levels of job satisfaction and life satisfaction enhancing performance in both domains as suggested by W-HR model.

### Agile leadership and inter-role conflicts (work–family and family–work conflicts)

Agile companies and organizations that are adept at anticipating change and adapting to it in ways that efficiently handle stakeholder and technical complexity are the key to sustained success. To develop agile organizations, agile leaders are vital (Joiner and Josephs, 2007). The direction on what to do, what to anticipate, and how to respond comes from leaders. Strong, composed, reliable leadership is more necessary than ever in tumultuous and uncertain times). It is the capacity to function well as a leader in situations of great complexity and quick change (Joiner and Josephs, 2007). The research described it as the type of leadership that exemplifies actions that add value, lower levels of waste associated with ideas and unproductive connections, and foster high levels of cooperation (Mollet, 2021).

The COVID-19 epidemic has brought up unusual circumstances that call for novel ways to leadership practices. It began as a health emergency before spreading fast, paralyzing the worldwide economy, social scene, education, and health care systems (Thu et al., 2020). The “suspended life without suspension” changed how people behaved (Hong et al., 2021). Every aspect of people’s personal and professional lives has changed significantly (Pokhrel and Chhetri, 2021). When a crisis brings issues for leaders, the same brings anxiety and stress for employees as well, often resulting in high turnover rates (Eichenauer et al., 2022). Many administrators in the education sector underestimate the risk of occupational stress, especially those new to the profession (Boyatzis et al., 2005). Among individuals, quarantine and social isolation caused many acute and chronic psychopathological issues, including stress, anxiety, depression, nervousness, and insomnia. These stressors triggered chronic work and home conflicts (Antino et al., 2022). The recent unprecedented telework arrangements brought challenges as both employees and leaders were not prepared to handle the inadequacy of home environments, severe childcare lapses, lack of technological competencies, lack of information, lack of teaching infrastructures at home, parallel management of families and work, internet connectivity, and other issues (Pokhrel and Chhetri, 2021; Eichenauer et al., 2022) causing stress to take over.

Family–work conflict is one of the several outcomes that can be defined as “a chronic inter-role stressor that captures the process whereby the family role detracts from the time, attention, and performance of the work role” (Badawy and Schieman, 2020). The boundary fading between work and family and pushing to shift work at home disturbed the balance and resulted in inter-role conflicts more easily. In connection with family–work conflict, the second most relevant outcome of this scenario in the VUCA world happens to be the work–family conflict which can be defined as “a form of inter-role conflict in which the role pressures from the work and family domains are mutually incompatible in some respect”. Work–family conflict is a type of inter-role conflict in which the demands of the roles in the work and family spheres are, in some ways, contradictory (Greenhaus and Beutell, 1985). The dual stressors, i.e., work and family together, posed a challenge, especially for education sector employees. Teachers had to educate other children (job demands) as well as their own at the same time (family demands), which caused imbalance (Hong et al., 2021). The ability to allocate, reallocate, and align resources according to work and family commitments is a challenge. The execution of parental duties, for instance, could be hampered by dedicating too many resources to work. The requirements of the work domain hamper the family’s needs. On the other hand, the achievement of their work-related activities is hampered by the excess of parental resources (Karakose et al., 2021).

Work–family conflict model presented by Frone et al. (1992) still dominates the literature. The model is based on the hypothesis that if people’s problems at work begin to interfere with their capacity to fulfill their family commitments, then these unfulfilled obligations may interfere with their ability to perform their daily tasks at work. On the other hand, people’s ability to function at home may start to suffer when their concern over family obligations starts to prevent them from performing their obligations at work (Karakose et al., 2021).

The dynamic accumulation model presented by Frese and Zapf (1988) shows that different individuals react to different stressors with varied symptoms and reactions in the context of feelings of job insecurity and work and family conflicts. A leader’s stress management skills, empathy, optimism, credibility, honesty, transparency, and interpersonal relationships play a strong role in mitigating the effects of stress among employees (American Psychological Association [APA], 2020). Research suggests that successful leaders in the times of crisis show both agentic and communal behaviors (Snyder, 2012). When a leader has agile leadership skills, he can be flexible and lift the team’s abilities, allowing them to contribute more to the organization’s development (Setiawati, 2021). Creating an environment that encourages everyone to approach problems creatively is the main responsibility of an agile leader. To achieve this, one must promote a sense of purpose shared by all and develop a capacity for quick learning. Agile leaders understand that this is not the time for conceit or exclusivism. However, all leaders know that they must learn how to influence their subordinates; however, few leaders consider doing so with individuals who do not directly report to them but have an impact on the future of their team (Hill, 2020).

Agile leaders are always involved with their employees, their families, customers, partners, suppliers, group members, staff, and other stakeholders in their business ecosystem. An agile leader listens and communicates. They spend much time interacting with the outside world (Setiawati, 2021). Consequently, while effective managers will exhibit agentic, task-oriented behaviors, and subordinates also consider social behaviors—such as demonstrating flexibility, social support, and sensitivity to work/family balance—to be more crucial for managers to exhibit in this disruptive environment along with agentic behaviors (Eichenauer et al., 2022). Agile leadership helps employees develop job embeddedness, which in turn helps enhance employee work–life balance and job satisfaction (Akkaya et al., 2020). Employees who relate to their workplace through effective leader–employee relations are better able to balance work and family obligations and are typically happier in their jobs (Mitchell et al., 2001; Safavi and Karatepe, 2018).

Agile leadership prioritizes useful ideas, values time, employs adaptable abilities, and tries to refrain from negative behaviors that drain resources (Shamani and Abbas, 2020). Organizations should focus on long-term solutions rather than quick fixes when addressing variables that could lead to work and family conflicts (Smith et al., 2022). Interference between work and family significantly and negatively affects both burnout and intention to quit, but work support from one’s top manager and leader moderates and reverses this influence (Jia and Li, 2022). Research repeatedly demonstrates a negative relationship between work–family conflict and support at work, particularly supervisor support (Anderson et al., 2002; Frye and James, 2004; Weale et al., 2021; Chang et al., 2022). Leaders and supervisors have mutual role expectations. It leads to a worldview that disregards organizational hierarchy in favor of a partnership in which both sides believe that their investment merits a commensurate response. As a result, the dynamic between the leader and the team affects a variety of work situations, including how well work and personal life are separated (Toscano et al., 2022). Keeping in view the literature cited above, this study hypothesizes that:

**H1:** Agile leadership practices have a direct negative relationship with family–work conflict.

**H2:** Agile leadership practices have a direct negative relationship with work–family conflict.

### Inter-role conflicts and individual satisfaction (life and job satisfaction)

A person’s work-life balance, work-family conflicts, job satisfaction, life contentment, and family satisfaction are all related to each other (Allen et al., 2010). Although role pressures, work–family conflict, and job satisfaction have been the focus of previous research studies (Beham et al., 2011; Ten Brummelhuis and Bakker, 2012), little is known about education sector employees’ work–family conflict and its effects during the severe public health emergency (Hong et al., 2021). Extant literature is available on the variables that impact levels of satisfaction among individuals investigating various demographic traits claiming that nature of work, working environment, and subjective factors as significant determinants (Çelik and Üstüner, 2018). Neugarten et al. (1961) were the first to define satisfaction as the state of meeting an individual’s needs, expectations, and wants. There are several aspects of satisfaction, out of which job satisfaction and life satisfaction have been the focus of attention for most researchers mainly because of their importance for individual- and organizational-level performance outcomes. According to Şirin and Döşyılmaz (2017), life satisfaction is a cognitive aspect of subjective well-being and an emotional state that results from contrasting what a person has accomplished in life with what they hope to acquire from it. Similarly, job satisfaction is another aspect of a pleasurable emotional condition brought on by an evaluation of one’s professional experiences on the job (Yousef, 2017).

In the work–family conflict model, Frone et al. (1992) assumed a cross-domain interaction, indicating that family-to-work conflict mostly affects the work domain. This presumption is justified by the notion that, despite its inception in one domain, the dispute contributes to issues in the other area. In turn, this other life domain’s well-being decreases as a result. A crucial outcome variable in the work–family conflict model is job satisfaction in the workplace (Amstad et al., 2011). Workplace well-being comprises more than just coping; it also includes having a fulfilling life, being optimistic, succeeding, and prospering (Bennett et al., 2017). As people embark on the work–life journey, it becomes crucial to consider one’s overall well-being and ability to strike a balance between professional and social responsibilities, as incompatibilities between these responsibilities have been shown to have minimal positive effects on one’s job and quality of life (Allen et al., 2000). People blame their circumstances at work, which in turn causes them to have unfavorable/favorable feelings toward their employment and affects their job satisfaction (Hong et al., 2021). Additionally, according to Diener (2000), contented individuals perform better at work and are more likely to record favorable outcomes. Working remotely created the possibility of family–work conflict when such balance was more difficult to attain (Galanti et al., 2021; Xiao et al., 2021), which effected the satisfaction levels of employees during pandemic (Toscano and Zappalà, 2020). Henceforth, there is a correlation between individual satisfaction and work and family-related conflict (Hong et al., 2021). Accordingly, based on the above literature review, it is hypothesized that:

**H3:** Family-to-work conflict has a direct negative relationship with job satisfaction.

**H4:** Family-to-work conflict has a direct negative relationship with life satisfaction.

**H5:** Work-to-family conflict has a direct negative relationship with job satisfaction.

**H6:** Work-to-family conflict has a direct negative relationship with life satisfaction.

### Agile leadership, inter-role conflicts, and individual satisfaction

Even though all leaders are aware of the need to develop their ability to influence those who report to them; however, few leaders understand that positive or negative spillovers from work also impact those who are indirectly related to the organizations, especially the families (Hill, 2020). An agile leader is more connected to its outside world with more flexibility and acceptance of issues related to internal and external stakeholders (Setiawati, 2021), exhibiting both communal and agentic behaviors (Snyder, 2012). They are more sensitive to helping employees balance work and family domains (Eichenauer et al., 2022), facilitating them to make a maximum contribution toward organizational development (Setiawati, 2021). It has been discovered that more organizational agility significantly improves performance, empowers employees, develops their competencies, fosters a customer-centric culture, and increases job satisfaction (Peters et al., 2020).

Irrespective of the fact that leaders try their best to mitigate the negative impacts of the VUCA world, the behaviors of individuals from different generations respond differently to achieving life and job satisfaction. According to Cran (2014), millennials will change jobs at least twenty times more than previous generations. If they see their supervisor or teammate acting like a friend, the millennials will be satisfied with their jobs and stay with the company. When they do not like their boss, millennials will quit their jobs without hesitation. Seeing these unique characteristics of millennials, the VUCA situation is a challenge for organizations, given that millennials will soon become much of the population in organizations or companies. Instead of working to meet their basic needs, millennials want to make a greater impact on both their lives and the lives of others (Myers and Sadaghiani, 2010). One of a supervisor’s main responsibilities is to support his or her subordinates socially as they hold positions in which they can regulate incentives, protection, encouragement, and inspiration for workers (Avolio and Sosik, 1999). Therefore, under these circumstances, agile leaders enhance job commitment and embeddedness, which in turn helps balance work and family, resulting in better job and life satisfaction (Akkaya et al., 2020). Effective leader–employee relationships enable employees to relate to their workplace, which often results in happier and more satisfied workers who are better able to balance work and family conflicts (Mitchell et al., 2001; Safavi and Karatepe, 2018). To increase job satisfaction and life satisfaction, managers can help staff by allowing work flexibility, fostering trust and autonomy, and fostering a supportive and low-stress work environment (Ahn, 2007). Hence, the following hypotheses have been developed based on the literature given above:

**H7:** Family-to-work conflict mediates the relationship between agile leadership and job satisfaction.

**H8:** Family-to-work conflict mediates the relationship between agile leadership and life satisfaction.

**H9:** Work-to-family conflict mediates the relationship between agile leadership and job satisfaction.

**H10:** Work-to-family conflict mediates the relationship between agile leadership and life satisfaction.

### Role stress

The direct and indirect relationships between work overload, stress, work–family conflict, and job satisfaction have been studied. Job satisfaction of employees working in the education sector is impacted by dual role overload, resulting in work and family conflicts in the context of the COVID-19 epidemic that is sweeping the globe, but this is still an area that needs further research (Hong et al., 2021). During the COVID-19 outbreak, in terms of teaching and learning continuity, students suddenly had to direct and regulate their learning and become digitally savvy; educators had to switch to online teaching overnight regardless of their comfort level, familiarity, or training in digital pedagogies; and parents had to morph into dual roles as parent–educators. This has put much psychological pressure on everyone because the transition to internet media necessitates a specific set of technical and pedagogical knowledge and skills. The steep learning curve and information overload, particularly for individuals unfamiliar or inexperienced with online learning and teaching, may have a detrimental influence on learners, who may feel demotivated and disheartened (Liyanagunawardena et al., 2013; Liyanagunawardena and Williams, 2021).

According to previous research (Beham et al., 2011; Ten Brummelhuis and Bakker, 2012; Armstrong et al., 2015; Lambert et al., 2019; Olaniyan, 2020), job overload and work-to-family conflict correlate positively. Research conducted on a sample of 337 Chinese preschool teachers, contested that work stress worsens work–family conflict (Gu and Wang, 2021). One’s judgment of life and job satisfaction is impacted by perceptions of work overload (Cao et al., 2020). In addition to facing difficulties at work, people are also coping with personal problems brought on by the epidemic, such as grief, anxiety, and social isolation (UCSF Weill Institute for Neurosciences, 2020). Employees surely find it more challenging to continue doing work-related tasks than before the epidemic, given this exceptional situation (Eichenauer et al., 2022). Individual contentment is impacted by either direct or indirect stressors associated with the teaching profession (Çelik and Üstüner, 2018). Teachers experience family role stress and work role overload when the workplace is moved into the home, especially if they have children (Xu, 2016). Parental stress during their in-home quarantine greatly increased during COVID-19 because, more specifically, mothers had more frequent and prolonged contact with their kids (Hong et al., 2021). Research suggests that lowering role conflict, role ambiguity, role overload, and work–family conflicts through raising engagement in decision-making and job variety might increase commitment and satisfaction (Griffin, 2001; Hogan et al., 2006; Lambert and Hogan, 2009). Keeping in view, the above literature following statements are hypothesized:

**H11:** Role stress acts as a moderator on the direct relationship between agile leadership and family–work conflict.

**H12:** Role stress acts as a moderator of the direct relationship between agile leadership and work–family conflict.

## Research methods

### Measures

This is a quantitative study, and the measures used were adopted from the extant literature available in this domain. To measure all the variables of the study a 5-point Likert scale was used (1 = strongly disagree and 5 = strongly agree). During phase 1 (From February 15, 2022 to February 28, 2022), to measure agile leadership, 32-item scale of agile leadership was developed by Akkaya et al. (2020); for measuring job satisfaction, 3-item scale was developed by Spector (1985); and for measuring life satisfaction, 5-item scale developed by Shin and Johnson (1978) was used. During the second phase of data collection (From April 01, 2022 to April 15, 2022), for measuring work–family conflict and family–work-conflict, 10-item scale was developed by Netemeyer et al. (1996), and for measuring role stress scale developed by Rizzo et al. (1970) and Imoisili (1986) was used.

### Sample and procedure

For this study, the data were collected through a self-administrative survey method, from male and female faculty working in higher education institutes of Pakistan in the Rawalpindi and Islamabad regions. Sampling unit included research assistants, lecturers, assistant professors, associate professors, and professors. The purposive sampling technique was used for selecting a sample for the study. Educational institutes were selected because during the COVID-19 public health emergency; the education sector was the second most affected segment of industries after the health care sector (Gautam et al., 2022; Nayak et al., 2022). The reason for selecting faculty members was the panic, pressure, and stress on them to manage the smooth conduct of the education delivery without disruption, which wreaked havoc on their personal and professional lives.

### Pilot study

A pilot test was done prior to administering the surveys to analyze the contextual validity of the constructs. Twenty questionnaires were distributed among the faculty members of two universities. After analyzing the results, a high intercorrelation was detected among the items of family–work conflict and work–family conflict. To reduce this high intercorrelation, a focus group was conducted with three HR and two research professors. Upon their suggestions, three items from both variables were removed (showing high intercorrelation), and only two items from each variable were retained, which were showing better impact.

### Common methods variance

To investigate common methods variance, Harman’s single-factor test (Gannon et al., 2021) was conducted. The highest portion of variance explained by a single factor was 29.36% (threshold 40%), while the main factors of the study explained 85.39% of the cumulative variance. A common method factor was used in the structural model by using an unmeasured method factor approach (Liang and Chia, 2014). This showed that the average variance was 67%, and the average method-based variance was 1.4%, displaying a ratio of 48:1. Hence, according to Hair et al. (2017), CMV is not a concern for this research. For reducing social desirability bias, (i) before administering the questionnaire, participants were informed about voluntary participation and data confidentiality terms, and a code specific to them was assigned, (ii) during questionnaire design phase, dependent and independent variables were placed in separate sections and were distributed in different phases.

### Data collection

Two questionnaires were administered 1 month apart with the assistance of volunteers and HR departments of educational institutes. The survey forms were in Urdu, a procedure of back-to-back translation was adopted to convert scale items from English to Urdu with the help of two lecturers of the Urdu department, which were later back-translated by two professors of English.

During the first phase (From February 15, 2022 to February 28, 2022), demographic information and data for agile leadership, job satisfaction, and life satisfaction were collected. While information regarding work–family conflict, family–work conflict, along with the moderating effect of role stress, was collected in the second phase (From April 01, 2022 to April 15, 2022).

### Descriptive statistics

During the two phases, a total of 450 questionnaires were distributed. As suggested by Bougie and Sekaran (2019) for a quantitative study, a sample of more than 30 and less than 500 is appropriate; 387 filled questionnaires were received back. Out of these, only 362 were used for final data analysis, and the rest were excluded because of their insufficiency in fulfilling reliability and validity criteria. The response rate of the study is 80.44%. Based on the demographic information, 51% of the participants were females and 49% were males. The majority (35.66%) of the participants were aged between 35 and 50 years of age, 53.04% of the respondents were working as lecturers, were unmarried (53.8%), and they were working for more than 5 years with the current organization (50.29%). Moreover, most of the participants were from private sector higher educational institutes (64.64%). Descriptive statistics are presented in Table 1 below.

**TABLE 1:** Descriptive statistics.

Demographics	Characteristics	Frequency	Percentage (%)	Skewness	Kurtosis
Gender	Male	177	49%	0.062	0.563
	Female	185	51%		
	**Total**	**362**	**100**		
Age (years)	20–34	117	32.31	0.308	0.270
	35–50	129	35.66		
	51–64	97	26.78		
	65–80	19	5.25		
	**Total**	**362**	**100**		
Designation	Professor	17	4.69	0.541	0.271
	Associate Professor	32	8.84		
	Assistant Professor	94	25.97		
	Lecturer	192	53.04		
	Research Assistant	27	7.46		
	**Total**	**362**	**100**		
Marital status	Married	167	46.2	0.236	0.221
	Unmarried	195	53.8		
	**Total**	**362**	**100**		
Tenure (years)	Less than a year	76	20.99	0.426	0.245
	1–5 years	104	28.72		
	More than 5 years	182	50.29		
	**Total**	**362**	**100**		
Sector	Public	128	35.36	0.231	0.310
	Private	234	64.64		
	**Total**	**362**	**100**		

*n* = *362*. Bold values represent the total responses and the total of the percentage.

## Results and findings

### Confirmatory composite analysis

To confirm the validity and reliability of the study scale, confirmatory composite analysis (CCA) is used (Henseler et al., 2014). Table 2 below shows the indicator loadings of all the study variables. Results confirm that all the indicator loadings are above the threshold level of 0.5 (Hair et al., 2020), ranging from 0.672 for AgileL 5 to 0.920 for LS3. One item RS5 has a low indicator value (0.419); however, it is retained for analysis. According to Hair et al. (2020), items with indicator loadings falling within the range of 0.40 and 0.70 should be considered for deletion only if deleting the items increase the composite reliability (CR) and average variance extracted (AVE) levels, affecting content validity of construct. As the CR and AVE values had shown no significant improvement, hence, no item was deleted at this stage. One item RS9 had an outer loading of 0.363 that was deleted because of a validity compliance issue.

**TABLE 2:** Indicator loadings.

	AgileL	FWC	JS	LS	RS	WFC
AgileL 1	0.761					
AgileL 10	0.719					
AgileL 11	0.682					
AgileL 12	0.734					
AgileL 13	0.677					
AgileL 14	0.714					
AgileL 15	0.802					
AgileL 16	0.707					
AgileL 2	0.747					
AgileL 3	0.789					
AgileL 4	0.697					
AgileL 5	0.672					
AgileL 6	0.692					
AgileL 7	0.719					
AgileL 8	0.795					
AgileL 9	0.754					
AgileL 10	0.742					
AgileL 11	0.691					
AgileL 12	0.711					
AgileL 13	0.752					
AgileL 14	0.810					
AgileL 15	0.791					
AgileL 16	0.689					
AgileL 17	0.722					
AgileL 18	0.687					
AgileL 19	0.776					
AgileL 20	0.842					
AgileL 21	0.786					
AgileL 22	0.757					
AgileL 23	0.677					
AgileL 24	0.779					
AgileL 25	0.767					
AgileL 26	0.698					
AgileL 27	0.833					
AgileL 28	0.721					
AgileL 29	0.797					
AgileL 30	0.743					
AgileL 31	0.684					
AgileL 32	0.789					
FWC1		0.843				
FWC2		0.911				
JS1			0.798			
JS2			0.895			
JS3			0.695			
LS1				0.689		
LS2				0.798		
LS3				0.920		
RS1					0.751	
RS10					0.817	
RS11					0.737	
RS12					0.736	
RS13					0.764	
RS14					0.768	
RS15					0.833	
RS2					0.752	
RS3					0.695	
RS4					0.757	
RS5					0.419	
RS6					0.625	
RS7					0.655	
RS8					0.707	
WFC1						0.893
WFC2						0.829

*n* = *362*.

**FIGURE 2:**
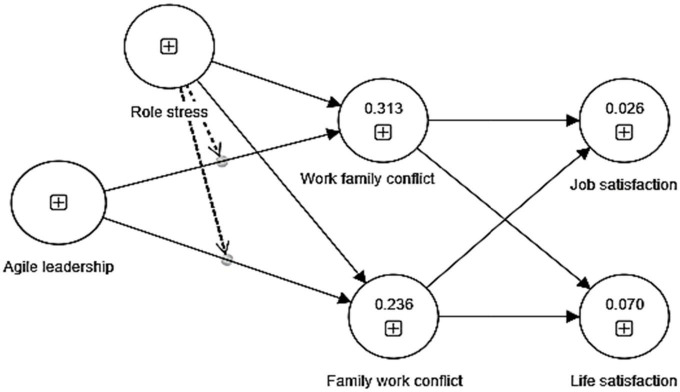
Measurement model.

The measurement model in Figure 2 below shows the measurement model used for this study.

Table 3 below shows the internal consistency measures of the study variables. The results show that the measures used are internally consistent and reliable. All the measures are above the threshold level of 0.70. The values of CA range from 0.714 for job satisfaction to 0.909 for agile leadership. The values of rho_A range from 0.742 for family–work conflict to 0.916 for agile leadership. Consequently, the values of CR range from 0.841 for job satisfaction to 0.921 for agile leadership. Moreover, the AVE values confirm the convergent validity of the measures used, as all the AVE values are above the threshold level of 0.5 (Hair et al., 2020). This confirms that all the items share high levels of interscale correlations conforming to standards of convergent validity.

**TABLE 3:** Internal consistency reliability.

	CA	rho_A	CR	AVE
Agile leadership	0.909	0.916	0.921	0.628
Family–work conflict	0.708	0.742	0.871	0.771
Job satisfaction	0.714	0.743	0.841	0.640
Life satisfaction	0.765	0.787	0.755	0.727
Role stress	0.878	0.886	0.892	0.667
Work–family conflict	0.755	0.776	0.852	0.742

*n* = *362*.

**FIGURE 3:**
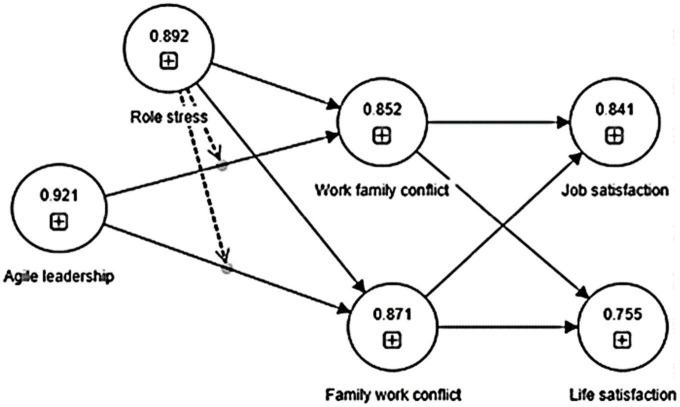
Reliability model. *The values within the variable show composite reliability of the constructs of the study.

For measuring discriminant validity, Fornell–Larcker and Hetro-Trait Mono-Trait criteria are used. The results in Table 4 below show that for the Fornell–Larcker criterion, all the diagonal values are greater than all the non-diagonal values. Moreover, all the HTMT values are below the threshold level of 0.90, establishing the discriminant validity of the scales used and determining that each scale measures dissimilar traits. Consequently, the complete bootstrap tests show that confidence intervals at a minimum of 2.5% and a maximum of 97.5% do not contain zero (Hair et al., 2020), which further confirms the discriminant validity. Figure 3 below shows the reliability model of variables used for the study.

**TABLE 4:** Discriminant validity measures.

Fornell–Larcker							HTMT					
	AgileL	FWC	JS	LS	RS	WFC	AgileL	FWC	JS	LS	RS	WFC
AgileL	**0.654**											
FWC	0.481	**0.878**					0.564					
JS	0.176	0.201	**0.801**				0.251	0.112				
LS	0.328	0.107	0.430	**0.654**			0.343	0.143	0.707			
RS	0.376	0.242	0.450	0.448	**0.606**		0.328	0.241	0.553	0.743		
WFC	0.552	0.228	0.154	0.270	0.231	**0.861**	0.694	0.349	0.219	0.258	0.242	

*n* = *362*. Diagonal bold values represent Fornell Larcker criterion values which must be higher from all other values in respective columns.

### Structural model

**FIGURE 4:**
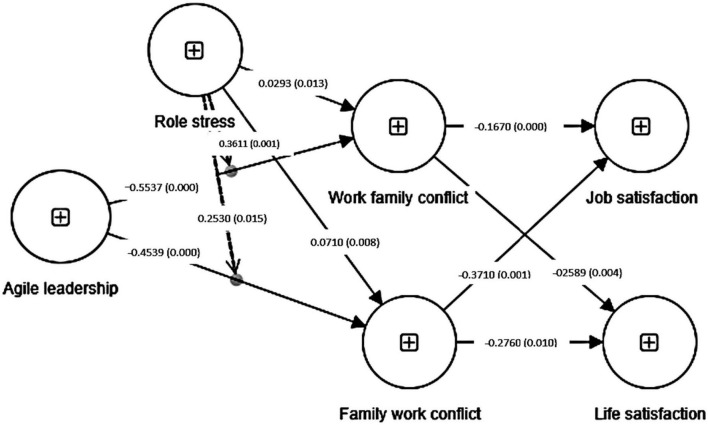
Structural model.

Figure 4 below shows the structural model of the study.

For testing the hypothetical model, the structural model assessment was performed through bootstrapping using 5000 samples. No multicollinearity was established through collinearity statistics as all the values were below the threshold level of 5. Table 5 below shows the results of structural model assessment. The estimates of direct relationships among variables show that agile leadership has a significant negative relationship with family–work conflict (FWC) H1 (β = −0.4539, *t*-value = 9.4167, *p-*value = *0.0000*) and work–family conflict (WFC) H2 (β = −0.5537, *t*-value = 10.4626, *p-*value = *0.0000*). FWC has a significant negative relationship with job satisfaction (JS) H3, (β = −0.3710, *t*-value = 8.6111, *p-*value = *0.0012*) and life satisfaction (LS) H4 (β = −0.2760, *t*-value = 5.6029, *p-*value = 0.0106). Similarly, WFC has significant negative relationship with JS (β = −0.1670, *t*-value = 2.5562, *p-*value = 0.0006) H5 and LS (β = −0.2589, *t*-value = 4.8564, *p-*value = 0.0046) H6. Moreover, all the values of bias-corrected confidence interval do not contain zero within, which establishes the stability of path coefficients.

**TABLE 5:** Direct relationships and mediation analysis.

Direct relationships	Hypotheses	Accepted/Not accepted	β	*t-*values	*p-*values	Bias Corrected CI	
						*2.5%*	*97.5%*
AgileL -> FWC	H1	Accepted	-0.4539	9.4167	0.0000	0.3550	0.5431
AgileL -> WFC	H2	Accepted	-0.5537	10.4626	0.0000	0.4499	0.6477
FWC -> JS	H3	Accepted	-0.3710	8.6111	0.0012	0.1352	0.2257
FWC -> LS	H4	Accepted	-0.2760	5.6029	0.0106	0.1284	0.1784
WFC -> JS	H5	Accepted	-0.1670	2.5562	0.0006	0.0246	0.2654
WFC -> LS	H6	Accepted	-0.2589	4.8564	0.0046	0.3090	0.3766

**Mediation analysis**	**Hypotheses**	**Accepted/Not accepted**	**β**	***t-*values**	***p-*values**	**Bias Corrected CI**	
						***2.5%***	***97.5%***

AgileL -> FWC -> JS	H7	Accepted	0.1598	9.6102	0.0017	0.1048	0.3971
AgileL -> FWC -> LS	H8	Accepted	0.1160	7.5989	0.0093	0.0562	0.0852
AgileL -> WFC -> JS	H9	Accepted	0.1020	2.5142	0.0112	0.0029	0.1533
AgileL -> WFC -> LS	H10	Accepted	0.1361	4.8707	0.0014	0.1460	0.2211

*n* = *362*.

Mediation analysis for the specific indirect relationship of AgileL -> FWC -> JS shows that family–work conflict partially mediates the direct relationship of agile leadership with job satisfaction, H7 (β = 0.1598, *t*-value = 9.6102, *p-*value = 0.0017). For AgileL -> FWC -> LS, the results show that FWC also partially mediates the direct relationship of agile leadership with life satisfaction H8 (β = 0.1160, *t*-value = 7.5989, *p-*value = 0.0093). For AgileL -> WFC -> JS, the results show that work–family conflict mediates the direct relationship of agile leadership and job satisfaction H9, (β = 0.102, *t*-value = 2.5142, *p-*value = 0.0112). Similarly, for AgileL -> WFC -> LS, mediation analysis indicates that work–family conflict also mediates the direct relationship of life satisfaction H10 (β = 0.136, *t*-value = 4.8707, *p*-value = 0.0014). The values of bias-corrected confidence intervals establish that mediational paths are stable and significant.

To estimate the significance of the moderation effect of role stress on the direct relationship of agile leadership with family–work conflicts and work–family conflicts, bootstrapping procedure with 5,000 iterations was performed. Results of moderation in Table 6 below reveal that role stress significantly moderates the direct relationship of agile leadership with family–work conflict H11 (β = 0.2530, *t*-value = 10.0185, *p*-value = 0.0152) and work–family conflicts H12 (β = 0.3611, *t*-value = 17.8872, *p*-value = 0.0012). The direct relationship of agile leadership with FWC and WFC shows a negative relationship showing that the increasing levels of agile leadership practices help reduce family and work conflicts. However, when the role stress increases, the direction of the predicted direct relationship changes, which signifies that as the role stress increases, the levels of FWC and WFC increase as well. This establishes the significance of the moderating impact of role stress.

**TABLE 6:** Moderation analysis.

Moderation effect	Hypotheses	Accepted/Not accepted	β	Simple effect	*t-*values	*p-*values	Bias-Corrected CI	
							*2.5%*	*97.5%*
RS*FWC -> FWC	H11	Accepted	0.2530	0.0710	10.0185	0.0152	0.0944	0.1158
RS*WFC -> WFC	H12	Accepted	0.3611	0.0293	17.8872	0.0012	0.0025	0.2160

*n* = *362*.

The blindfolding procedure to determine the predictive ability of the study model is used with an omission distance of 7. Results in Table 7 below show construct cross-validity redundancy. All the Q^2^ values are well above zero, signifying that the model is predictive of the hypothesized relationships. Family–work and work–family conflicts show medium levels of predictability, while job satisfaction and life satisfaction show small levels of predictive model ability. This establishes that these variables can be kept in the model having significant levels of predictive ability to predict endogenous variables.

**TABLE 7:** Blindfolding.

Variables	SO	SSE	Q^2^ (=1 − SSE/SSO)	Remarks
Agile leadership	4896	4896		
Role stress	4590	4590		
Family–work conflict	612	509.3736	0.2677	Medium
Work–family conflict	612	478.1058	0.2188	Medium
Job satisfaction	918	905.3963	0.0837	Small
Life satisfaction	918	910.8047	0.0978	Small

*n* = *362*.

## Discussion, conclusion, and implications

### Discussion

The COVID-19 pandemic has been the worst public health emergency witnessed by the people of this century, causing agony and turmoil around the globe (Mallah et al., 2021; Panneer et al., 2022). The initial impacts were thought to be limited to individual health only; however, as the periods of quarantining and expanse of disease spread increased, it was observed that impacts of this public health emergency are not just limited to health and are far beyond what was realized. Since its outbreak, organizations have been under siege by changing scenarios and emerging challenges on the fabric of the world’s business platforms (Fitzpatrick, 2022). More so, the need for effective leadership has never been so fervently understood as in this VUCA world scenario (Botbyl, 2022). These testing times were the incubator for testing agility at organizational and individual levels (Frare and Beuren, 2021). Numerous studies have shown that the teaching profession is characterized by a high rate of discomfort, dissatisfaction, an increased workload, and stress. This pandemic’s problems have gotten worse, making it challenging for teachers compelling them to teach online without any significant support. COVID-19 caused significant, ongoing unfavorable modifications in teaching methods for instructors all around the world (Guoyan et al., 2021). Before COVID-19, the United Nations Development Programme [UNDP] (2019), rates Pakistan at 152 out of 189 nations in the UNDP’s Human Development Index (HDI) rating. Sadly, Pakistan has not shown improvement in important educational metrics including literacy rate, gross enrollment ratio, and investment on education (Guoyan et al., 2021), and the advent of pandemic made it only worse. However, leadership was the differentiating factor in these testing times.

Agile leadership research and its implications have been the focus of attention since the COVID-19 outbreak. The current study has investigated how agile leadership practices influence employees’ job satisfaction and life satisfaction through the mediational effects of work–family and family–work conflicts. This study also explored the relationship of role stress as a moderating variable on the direct relationship between agile leadership and inter-role conflicts. As hypothesized, the study found a significant negative relationship between agile leadership and work–family conflicts and family–work conflicts, which are also supported by studies conducted by Akkaya et al. (2020) and Jia and Li (2022). In Pakistani context, research suggests that academic and adaptive leadership helped implement change and develop higher education institutes (Mukaram et al., 2021). For instance, in Pakistan, HEC is dedicated to making e-learning success in the wake of the COVID-19 pandemic, COVID-19-specific policies and procedures and emergent levels of support helped leadership in educational institutes facilitate students and employees at the same time (Mumtaz et al., 2021). Similarly, work–home resources model (W-HR) also supports the findings by explaining that contextual work resources improve family outcomes by augmenting the personal resources of employees. This help caters to the dual role demands of being a family person while working from home and vice versa (Ten Brummelhuis and Bakker, 2012). So, it can be justified that leadership agility in the times of crisis and emergency plays its role in managing inter-role conflicts.

Job satisfaction and life satisfaction are two critical job behaviors. Extant literature talks about the antecedents and outcomes of these aspects of satisfaction (Çelik and Üstüner, 2018). However, in this VUCA world situation during the COVID-19 pandemic, there is a huge gap in research in observing the two job behavioral outcomes under the changed world scenarios (Dima et al., 2021). The education sector, one of the hardest hit sectors, was like a boiling pot with evolutionary changes resulting in stress, depression, anxiety, dissatisfaction, incompetency, and non-willingness to perform (Tang et al., 2021). The “infodemic” in Pakistan resulted in economic instability, individual mental health issues, behavioral problems, and unskilled human capital flow toward industries (Haider et al., 2021). Faculty found it difficult to be flexible enough to become tech savvy and adapt to the changing situations. It was difficult for educational institutions to match the internal pace of change with the external pace of change (Mukaram et al., 2021). According to the W-HR model, as employees develop their resources such as elevated moods, better self-esteem, and positive emotional support, it has a significant impact on performance, as these resources help improve personal resources at work (Ten Brummelhuis and Bakker, 2012). So, this model supports the findings. When leaders are empathetic, support flexibility, and display cooperative behaviors, it is easier to manage inter-role conflicts, which in turn help employees provide meaning to work and enhance job satisfaction and life satisfaction (Grimes, 2022).

During the times of uncertainty like never before, the need to protect and assist family (family demands) and the need to be an effective employee (job demands) at the same time led to a confused workforce. The role stress increased as job demands became more and more ambiguous (Liyanagunawardena and Williams, 2021; Zheng et al., 2022). Employees lacked the ability to immediately switch to the online mode due to a declared education emergency ending up with “technostress” among Pakistani faculty members. The immediate shutdown of the hustle and bustles of life with ever so-active and busy quarantine life led to role conflicts with employees who were unable to allocate proper time and resources to work and family, resulting in an inability to separate the two while they were not (Mushtaque et al., 2022). This role overload and the increasing demands of managing work and family lead to lower job and life satisfaction levels. Intentions to quit jobs people loved were on an all-time high. The W-HR model supports the findings of this study as it suggests that context-specific variables influence employee behaviors and their outcomes (Ten Brummelhuis and Bakker, 2012).

### Theoretical implications

This study is a novel addition to the empirical literature available on employee satisfaction in the wake of COVID-19 pandemic VUCA world scenario specifically in Pakistani context, which was not changed ready as per its current economic state and the quality of the infrastructure available. The suggested mechanism is not studied earlier that how agile leadership behaviors can help manage two critical types of resultant role conflicts, i.e., work–family and family–work conflicts, specifically when the world came to a halt and the families were together yet they were the most distant because of the role overload, ambiguous work boundaries, and conflicting roles. Employees once passionate about their jobs as faculty were thinking of quitting because of the reason they were not able to handle the pressures and resultant lack of meaningfulness in job and life. So, this study provides valuable insight into how agile leaders in the times of crisis can manage employee behaviors and the resultant outcomes. Moreover, the education sector is considered as the most contributing sector from the strategic point of view. This study highlights the issues faced by teachers and how the leaders in this domain handled employee issues, helped manage work and family life, and retained their faculty in the times of crises, which benefited the organizations and employees in broader strategic contexts.

### Practical implications

Agile leadership behaviors are positive supervisory behaviors that promote flexibility, adaptability, innovation, and creativity. Leaders’ social and communal sensitivity in times of crisis helps realize the importance of leadership agility within organizations. This study will help organizations understand how leadership ability can mitigate the effects of uncertainty and risk affecting employees’ personal and professional lives. The educational institutes, in addition to the health sector, have witnessed transformational changes and gave new meaning to learning and development for both students and faculty. So, this study is important for higher educational institutes to understand the importance of managing work and family conflicts by emphasizing that managing inter-role conflict is not just the duty of employees, leader’s role is of critical importance in uncertain times like these.

### Limitations and future directions

This study has collected data from faculty of higher education institutes which played a very critical role in helping students maintain education continuity; however, to ensure the generalizability of the results of this study, future researchers may consider testing the model in the health care sector. Moreover, this study is conducted after the lockdowns are lifted and employees have returned to normal working conditions. Comparative studies can be conducted on the role of agile leaders in health care and education sector institutes. Future researchers may also consider the impacts of role ambiguity, role conflict, and role overload on work–family and family–work conflicts separately. Future researchers may also consider longitudinal and experimental research designs to retest the results of this study. Personal factors such as employee self-efficacy, proficiency in adapting to change, and fear of the COVID-19 pandemic may also be considered antecedents of inter-role conflicts for future research in this domain.

## Data availability statement

The raw data supporting the conclusions of this article will be made available by the authors, without undue reservation.

## Ethics statement

Ethical review and approval was not required for the study on human participants in accordance with the local legislation and institutional requirements. The patients/participants provided their written informed consent to participate in this study.

## Author contributions

SA conceived the idea of this manuscript and performed the data analysis. SA and AW drafted the original manuscript. SA and AAf collected data for the study. SA and KK contributed in writing methodology. SA, AAf, and AAd contributed in discussion and conclusion sections. AW supervised the writing stage. KK, AAf, and AAd helped in proofreading and revising the manuscript. All authors contributed to the article and approved the submitted version.
